# Identification and characterization of *Schizosaccharomyces pombe* splicing mutants

**DOI:** 10.17912/micropub.biology.002096

**Published:** 2026-03-17

**Authors:** Laurel L. Nicholas, Fang Suo, Sarah M. Hanna, Li-Lin Du, Kathleen L. Gould

**Affiliations:** 1 Department of Cell and Developmental Biology, Vanderbilt University School of Medicine, Nashville, TN, US; 2 National Institute of Biological Sciences, Beijing, Beijing, BJ, CN

## Abstract

Pre-mRNA splicing is carried out by the spliceosome, a dynamic complex of five small nuclear ribonucleoprotein particles (snRNPs). Several genetic screens have been conducted in *Schizosaccharomyces pombe* to identify pre-mRNA splicing mutants and spliceosome components *.* However, some pre-mRNA splicing mutants have yet to be assigned to a gene and in certain cases, the mutations within genes have not been identified and phenotypes compared. Here, we have identified new mutations in the U4/U6.U5 tri-snRNP component *dim1* and assigned *prp6* and *prp7* mutants to *snu13* and *brl1* , respectively, revealing roles for these factors in pre-mRNA splicing.

**Figure 1. Sequence analysis of pre-mRNA splicing mutants.**
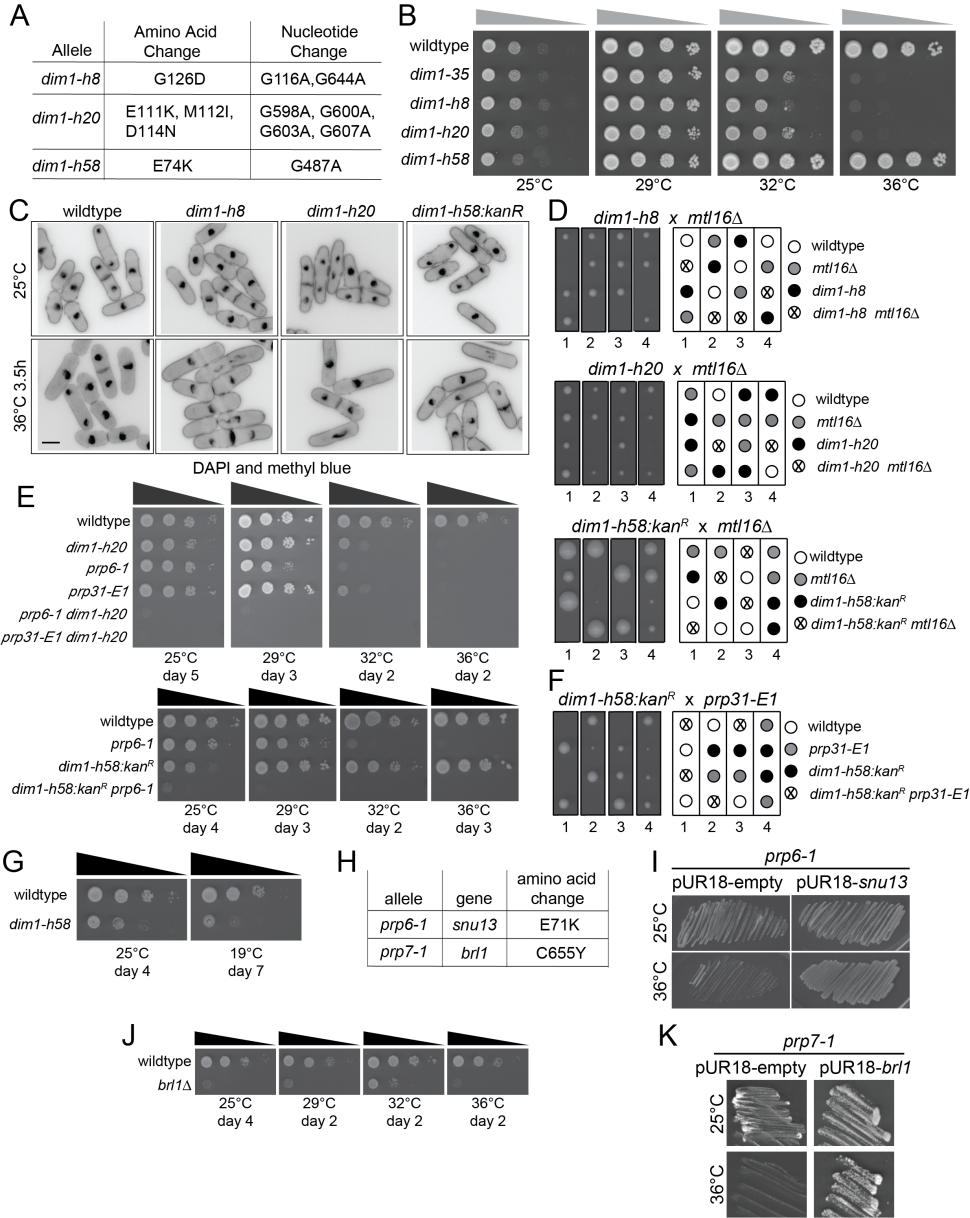
(A) The mutations encoded by each *dim1* allele are listed. (B, E, G, and J)&nbsp;The indicated strains were grown in liquid YE media at 25°C until they reached mid-log phase and then adjusted to OD = 0.20. Next, 10-fold serial dilutions were made and 2.5 µL of each was spotted on YE agar plates and incubated at the indicated temperatures for 2-7 days prior to imaging. Images of all plates shown in panel B were acquired after less than 3 days of growth, accounting for the difference in wildtype growth at 25˚C compared to other panels. (C) The indicated strains were grown at 25˚C and shifted to 36˚C for 3.5 hours. Samples were collected at both temperatures and cells were fixed and stained with DAPI and Methyl Blue before imaging. (D and F) Representative tetrads incubated on YE plates at 25˚C from the indicated cross and schematics of relevant genotypes. (H) The responsible gene and mutations encoded by each allele are listed. (I and K) *prp6-1* (I) or *prp7-1* (K) cells containing the indicated plasmids were streaked to the indicated temperatures and incubated for 3-5 days.

## Description

&nbsp;Pre-mRNA splicing requires the activity of the spliceosome, a large and dynamic complex comprised of dozens of protein components (Wilkinson et al., 2020). In *S. pombe* , almost half of the ~5134 protein coding genes contain one or more introns in their pre-mRNAs that require spliceosome-mediated excision (Carme et al., 2026; Rutherford et al., 2024; Wood et al., 2012). This complexity which also includes the use of degenerate splice site sequences, exonic splicing enhancers, and SR proteins, makes *S. pombe* an attractive organism for the elucidation of pre-mRNA processing mechanisms (Fair and Pleiss, 2017).

Dim1 is an essential, highly conserved component of the U4/U6.U5 tri-snRNP (Berry and Gould, 1997; Gottschalk et al., 1999; Reuter et al., 1999; Stevens and Abelson, 1999; Zhang et al., 2000; Zhang et al., 1999). In a genetic screen designed to identify genes cooperating with Cdk1 to promote the G2/M transition, we isolated the *dim1-35* mutation because it reduced the restrictive temperature of *cdc2-D217N* (Berry and Gould, 1997). The *dim1-35* mutant, in which amino acid 126 is changed from G to D, is defective in pre-mRNA splicing (Carnahan et al., 2005), displays significant cell cycle defects (Berry and Gould, 1997), and is synthetically lethal with a mutant in the anaphase-promoting complex, *lid1-6* (Berry et al., 1999) and with loss of U6 snRNA m ^6^ A methyltransferase Mtl16 function (Willet et al., 2023). In the same screen used to isolate *dim1-35* , three additional *dim1* mutant alleles were obtained but not characterized. Here, we determine their mutations and examine their phenotypes and genetic interactions. We also identify the causative gene mutations in two previously unassigned pre-mRNA splicing mutants.

To determine what mutations were present in the previously uncharacterized *dim1* alleles, the open reading frame was amplified from each strain and sequenced. The *dim1-h8* strain contained the same amino acid change as did *dim1-35* (Berry and Gould, 1997) plus a nucleotide change within the second intron that is not at an intron junction or the splicing branch point. *dim1-h20* contained four mutations leading to three amino acid changes and the *dim1-h58* strain contained a single mutation resulting in an E74K change ( Figure 1A ). The range of growth was determined for each strain by spotting at a variety of temperatures. The *dim1-h8* and *dim1-h20* mutants showed similar temperature-sensitivity to *dim1-35* and failed to form colonies at 36˚C ( Figure 1B ). In contrast, *dim1-h58* was not heat-sensitive ( Figure 1B ) and therefore to follow it in crosses, the *kanMX6* cassette was inserted after the *dim1* stop codon. DAPI and Methyl Blue staining showed that *dim1-h8* resembled *dim1-35* at the non-permissive temperature with a variety of chromosome segregation errors including chromatin bisected by septa (Berry and Gould, 1997) ( Figure 1C ). *dim1-h20* and *dim1-h58:kanR* cells appeared a bit elongated at 36˚C and the chromosomes were sometimes disorganized but cut cells were not observed ( Figure 1C ). Like *dim1-35* (Willet et al., 2023), all three previously uncharacterized *dim1* mutants were synthetically lethal with *mtl16∆* ( Figure 1D ). Also like *dim1-35, dim1-h20* displayed significant negative genetic interactions with *prp6-1* and *prp31-E1* ( Figure 1E ). Interestingly, despite a lack of heat-sensitivity on its own, *dim1-h58* also displayed a significant negative genetic interaction with *prp6-1* ( Figure 1E ) and was synthetically lethal with *prp31-E1* at 25˚C ( Figure 1F ). These genetic interactions prompted us to test whether *dim1-h58* was cold rather than heat sensitive. Indeed, we found that it was ( Figure 1G ).

Like Dim1 , Prp31 is a component of the U4/U6.U5 tri-snRNP and both *dim1-35* and *prp31-E1* show significant negative genetic interactions with *prp6-1* (Bishop et al., 2000; Willet et al., 2023). Interestingly, neither *prp6-1* nor *prp7-1,* among the first *S. pombe* temperature-sensitive mutants defective in pre-mRNA processing identified (Potashkin et al., 1998; Potashkin et al., 1989; Urushiyama et al., 1996), have been assigned to a gene. To learn what gene mutations gave rise to these two *prp* mutants, we crossed each of the two strains to wildtype, grew 8 colonies of wildtype and 8 colonies of mutant obtained from crossing to our laboratory wildtype strain, combined the 8 samples of each at equal cell concentrations, and the genomes of the two samples were sequenced and analyzed. A comparison of the mutant sequences to that of wildtype indicated that the *prp6-1* strain carries an E71K substitution in the *snu13* open reading frame (G211A) ( Figure 1H ). Snu13 is required for both pre-mRNA splicing and pre-rRNA splicing in *Saccharomyces cerevisiae* and is a tri-snRNP component (Dobbyn et al., 2007; Dobbyn and O'Keefe, 2004) that in *S. pombe* co-purifies with Dim1 (Carnahan et al., 2005). Amplifying and sequencing the *snu13* gene from *prp6-1* cells confirmed the presence of this mutation. The *psc3* gene is immediately adjacent to *snu13* on chromosome I and we found that no wildtype progeny were recovered from a cross between *prp6-1* and *psc3-1T:kanR* (Nonaka et al., 2002) in a plate of tetrads. Further, plasmid-expressed *snu13 ^+^* was able to rescue *prp6-1* temperature-sensitivity ( Figure 1I ). Taken together, we conclude that the Snu13 E71K substitution is responsible for the *prp6-1* phenotype.

Two candidate mutations were identified in the *prp7-1* strain relative to wild type: *mdn1-D4125N* (G12373A) and *brl1-C655Y* (G1964A). We focused our attention on *brl1* because its homolog in *S. cerevisiae* , *BRE1* , has been linked to pre-mRNA splicing (Moehle et al., 2012) through its role in histone H2B ubiquitination (Herissant et al., 2014; Moehle et al., 2012). In contrast, there is no evidence that Mdn1 plays a role in pre-mRNA splicing. Amplifying and sequencing the *brl1* gene from *prp7-1* cells confirmed the presence of the *brl1-C655Y* mutation. The *bmt5* gene is closely linked to *brl1* on chromosome III (Lock et al., 2018) and we found no recombinants between *bmt5∆::kanR* and *prp7-1* in 10 complete tetrads. Although *brl1∆* is not an essential gene, *brl1∆* cells were reported to be slow growing and highly elongated with an increased septation index (Tanny et al., 2007; Zofall and Grewal, 2007). A similar elongated, hyphal-like phenotype was also noted for *prp7-1* cells after shift to the non-permissive temperature (Potashkin et al., 1998). We verified that the *brl1∆* strain grows slowly and also determined that it is unable to form colonies at all on YE plates at 36˚C ( Figure 1J ). Finally, plasmid-expressed *brl1 ^+^* was able to rescue the temperature-sensitivity and also the slow growth at 25˚C of *prp7-1* ( Figure 1K ). We conclude that the *prp7-1* pre-mRNA processing phenotype is due to the mutation in *brl1* .

In sum, our data provide evidence for the cooperation of Dim1 , Prp31 , and Snu13 in spliceosome activation. Further, by identifying the mutations in the *prp6-1* and *prp7-1* strains we firmly link both Snu13 and Brl1 to the process of pre-mRNA splicing, enhancing the toolkit of reagents with which to investigate mechanisms of this critical process in *S. pombe* .

## Methods

Yeast methods

*S. pombe* strains were grown in yeast extract (YE) or Edinburgh minimal medium (EMM) supple­mented with appropriate amino acids with appropriate supplements and standard&nbsp; *S.&nbsp;pombe* &nbsp;mating, sporulation, and tetrad dissection techniques were used for backcrossing, outcrossing, and to construct new strains (Forsburg and Rhind, 2006; Moreno et al., 1991). EMM with 5 µg/ml thiamine was used to repress expression of murine *dim1* from the *nmt1* promoter.

To construct&nbsp; *dim1-h58:kanMX6,&nbsp;* PCR was utilized to amplify a sequence containing the kanamycin resistant gene in a pFA6 cassette (Bahler et al., 1998). Through lithium acetate transformation (Keeney and Boeke, 1994), this sequence was inserted after the final stop codon of the&nbsp; *dim1-h58&nbsp;* gene. Colonies were selected by replication onto YE plates containing G418 (Geneticin, 100 µg/mL, Thermo Fisher Scientific; cat# 11811031), and correct marking of the *dim1* allele was confirmed through whole-cell PCR.

For growth assays, strains were grown overnight in YE at 25°C to OD _595_ =0.1-0.8. Cells were then adjusted to OD _595_ = 0.20 and then diluted tenfold thrice. 2.5 μL of each dilution were spotted onto YE plates and grown at the indicated temperatures for several days. Each growth assay was performed twice.

Molecular biology methods

A PCR product was generated from each *dim1* allele using an oligonucleotide 59 bp upstream of the start site (GTATATTGTGTGACTTACATATCTACA) and 243 bp downstream of the stop codon (GCAGTAATCATGTTCATGC) (Integrated DNA technologies), purified, and sent for sequencing. To construct plasmids containing&nbsp; *brl1 &nbsp;* and&nbsp; *snu13 ,&nbsp;* PCR was used to amplify sequences of&nbsp; *brl1 &nbsp;* and&nbsp; *snu13 &nbsp;* from wildtype genomic DNA that *&nbsp;* included 300 bp flanks upstream and downstream of the coding sequence. These fragments were each cloned into the BamHI site of pUR18 (Barbet et al., 1992) using Gibson assembly (Gibson et al., 2009). Subsequently, the pUR18- *brl1 &nbsp;* and pUR18- *snu13 &nbsp;* plasmids were transformed into&nbsp; *prp7-1&nbsp;* and&nbsp; *prp6-1,&nbsp;* respectively, using the lithium acetate transformation method (Keeney and Boeke, 1994), and colonies were selected on EMM plates lacking uracil. PCR products, plasmids, and whole genomes were sequenced by Plasmidsaurus using Oxford Nanopore Technologies &nbsp;(ONT) long-read sequencing.

ONT read mapping and variant calling

Whole-genome ONT sequencing reads were down-sampled to 30x depth coverage using seqtk v1.5-r133 (https://github.com/lh3/seqtk). Down-sampled reads were mapped to the reference genome with minimap2 v2.30-r1287 (https://github.com/lh3/minimap2) (Li, 2018). Variants were called using Clair3 v1.2.0 (https://github.com/HKU-BAL/Clair3) (Zheng et al., 2022) with the r1041_e82_400bps_sup_v500 model. Clair3-generated gVCF files were merged using GLnexus v1.4.1 (https://github.com/dnanexus-rnd/GLnexus) (Yun et al., 2021). Variants were annotated using SnpEff v4_3t (https://pcingola.github.io/SnpEff/) (Cingolani et al., 2012). Candidate mutations were defined as variants predicted to cause amino acid changes that were present in the mutant sample but absent in the wild-type sample. Detailed scripts, commands, and parameters used in this analysis are available at (https://github.com/fsnibs10/prp6_prp7).&nbsp;Whole-genome ONT sequencing reads have been deposited in the Sequence Read Archive (SRA) of the National Center for Biotechnology Information (NCBI) under BioProject accession PRJNA1428504 .

Microscopy methods

To visualize nuclei and septa, cells were grown overnight in YE to log phase at 25°C, shifted to 36.5°C for 3.5 hours and fixed in ice-cold 70% ethanol. Samples were then washed in phosphate-buffered saline three times and stained with 1 mg/mL Methyl Blue (Millipore Sigma) and 4',6-diamidino-2-phenylindole (DAPI). Single medial Z slices were obtained using a Zeiss Axio Observer inverted epifluorescence microscope with an AxioCam 503 mono camera and a Zeiss Plan Apochromat 63x oil (1.46 nA) objective. Representative images were formatted using ImageJ&nbsp;(Schindelin et al., 2012).

## Reagents

**Table:** 

Strain	Genotype	Source
KGY246	* ura4-D18 leu1-32 ade6-M210 h ^-^ &nbsp;&nbsp; *	Lab stock
KGY247	* ura4-D18 leu1-32 ade6-M210 h ^+^ &nbsp; *	Lab stock
KGY390	* dim1-35 ura4-D18 leu1-32 h ^-^ &nbsp;&nbsp; *	Lab stock
KGY1152	*dim1-h8* *ura4-D18 leu1-32 ade6-M21X h ^-^*	This study
KGY5712-2	* dim1-h8 ade6-M210 ura4-D18 leu1-32 h ^+^*	This study
KGY1153	*dim1-h20* *ura4-D18 leu1-32 ade6-M21X h ^-^*	This study
KGY1160	*dim1-h20* *h ^-^*	This study
KGY8680-2	* dim1-h58 ura4-D18 leu1-32 ade6-M210 h ^+^*	This study
KGY7226-2	* dim1-h58:kanMX6 ura4-D18 leu1-32 ade6-M210 h ^-^*	This study
KGY1301	*mtl16 ::ura4 ^+^ ura4-D18 leu1-32 ade6-M21X h ^-^*	Willet et al., 2023
KGY1844	*mtl16 ::ura4 ^+^ ura4-D18 leu1-32 ade6-M21X h ^+^* &nbsp;&nbsp;	Willet et al., 2023
KGY2457	*prp31-E1 ade6-M210* *leu1-32 ura4-D18* *h ^-&nbsp;&nbsp;&nbsp;&nbsp;&nbsp;&nbsp;&nbsp;^*	Bishop et al., 2000
KGY1877	* prp6-1 leu1-32 h ^-^* &nbsp;	Potashkin et al., 1998
KGY8434	* prp6-1 ura4-D18 h ^+^*	This study
KGY8678-2	*dim1-h20* *prp31-E1 ade6-M210* *leu1-32 ura4-D18* *h ^-^*	This study
KGY8679-2	*dim1-h20* *prp6-1 ade6-M210* *leu1-32 ura4-D18* *h ^-^*	This study
KGY7045-2	*dim1-h58:kanMX6 prp6-1 ade6-M210* *leu1-32 ura4-D18* *h ^-^*	This study
KGY7218	* prp7-1 leu1-32 h ^-^*	Potashkin et al., 1998
KGY7591-2	* prp7-1 ura4-D18 leu1-32 Ade6? h ^?^*	This study
KGY7671	* pcs3-1T:kanR ade6-M210 h ^-^*	Nonaka et al., 2002
KGY7216-2	* brl1∆::kanMX6 ade6-M210 ura4-D18 leu1-32 h ^+^*	Bioneer V2
KGY7546-2	* bmt5∆::kanMX6 ade6-M210 ura4-D18 leu1-32 h ^+^*	BioneerV2
